# Peripheral Artery Disease in Asian Ischaemic Stroke Patients—A Cross-Sectional Study

**DOI:** 10.3390/neurosci7030059

**Published:** 2026-05-15

**Authors:** Narayanaswamy Venketasubramanian

**Affiliations:** Raffles Neuroscience Centre, Raffles Hospital, Singapore 188770, Singapore; ramani_nv@rafflesmedical.com; Tel.: +65-6311-1111

**Keywords:** ischaemic stroke, peripheral arterial disease, non-lacunar infarction, Asian

## Abstract

Peripheral artery disease (PAD) is found in 10.9% of patients with ischaemic stroke (IS). This cross-sectional study was performed to investigate the prevalence of PAD and its risk factors among acute IS patients in Singapore. Patients admitted for IS were recruited. Data was collected on sex, age, body mass index (BMI), history of hypertension, diabetes mellitus (DM), hypercholesterolaemia, cigarette smoking, prior stroke (PS) and ischaemic heart disease (IHD). IS was classified as a lacunar infarct (LI) or non-lacunar infarct (NLI) based on neuroimaging. Carotid intima–medial thickening (IMT) and carotid plaques (CP) were determined by ultrasonography. The ankle–brachial Index (ABI) was calculated in both lower limbs; PAD was diagnosed if the ABI was ≤0.9 in any limb. The estimated sample size was 150 subjects. In total, 150 subjects were recruited; the mean age was 62.7 ± 10.2 years, 44.7% were female, and the mean BMI was 24.1 ± 4.1. A total of 63.3% reported hypertension, 42.7% DM, 30.0% hypercholesterolaemia, 38.0% smoking, 18.7% PS, and 6.0% IHD. A total of 30.7% had IMT, 77.3% had CP, and 8.0% had carotid stenosis ≥50%. LI occurred in 64.7%. PAD was diagnosed in 22.0% (95% CI 16.1–29.3). On univariate analysis, based on vascular risk factors alone, PAD was associated with age (*p* = 0.03), hypercholesterolaemia (*p* = 0.03), and IHD (*p* = 0.004). On logistic regression, PAD was only associated with IHD (aOR 6.42, 95% CI 1.25–32.84; *p* = 0.03). When IMT and CP were added to the model, the association with IHD remained (aOR 5.45, 95% CI 1.03–28.71; *p* = 0.045). When the results of neuroimaging were added, the association was only with NLI (aOR 2.78, 95% CI 1.09–7.14; *p* = 0.03). This study found a high prevalence of PAD among Asian patients with IS. It was associated with a non-lacunar infarction.

## 1. Introduction

### 1.1. Epidemiology of Stroke

Ischaemic stroke (IS) is a major cause of death and disability globally. In 2021, based on the Global Burden of Disease study, IS accounted for 3.6 million deaths (95% UI 3.2–3.9 million), 7.8 million incident cases (95% UI 6.7–8.9 million), 69.9 million prevalent cases (95% UI 64.8–75.0 million), and 70.4 million disability-adjusted life years lost (DALYs) due to stroke (95% UI 64.3–76.0 million) [1]. With the growth in absolute numbers, and the ageing of the world’s population from increased longevity, these numbers can only be expected to rise in the foreseeable future. In a large, case-controlled, 32-country international INTERSTROKE study that included 10,388 patients with IS, the modifiable risk factors for stroke included hypertension, diabetes mellitus (DM), apolipoproteins and smoking [2]. As for mechanisms causing IS, in INTERSTROKE, 44% of IS was due to small-artery occlusions leading to lacunar infarctions, while the remainder were non-lacunar infarctions due to various mechanisms, especially large-artery atherosclerosis [3].

### 1.2. Epidemiology of Peripheral Artery Disease

In 2019, an estimated 113 million individuals worldwide suffered from peripheral artery disease (PAD), accounting for 1.5 million DALYs lost [4]. PAD is part of the spectrum of atherosclerosis, where there is a structural change in the intima and media of medium- and large-sized arteries that leads to arterial thrombosis, which results in local arterial occlusion or artery-to-artery embolism with distal occlusion, a generalised disease process known as atherothrombosis/atheroembolism [5]. Atherosclerosis is a systemic polyvascular disease that affects multiple vascular beds—peripheral (limb) arteries, coronary arteries and the cerebrovascular circulation—by the same pathologic processes regardless of the vascular bed involved [6]. The risk factors for atherosclerosis include hypertension, DM, hyperlipidaemia and cigarette smoking [7], similar to IS [2]. However, there are differential effects of these risk factors, depending on the vascular bed, with hypertension affecting the cerebrovascular circulation more, smoking affecting the peripheral vasculature more, and diabetes mellitus affecting both circulation territories [8].

### 1.3. PAD Among Those with Disease in Other Arterial Beds

Patients with an existing atherosclerotic disease in one vascular bed are at high risk of having an ischaemic vascular event in the same or another vascular bed [9]. Among those with established cerebrovascular disease (CeVD), having concomitant PAD increases the risk of subsequent major atherothrombotic events. The Reduction of Atherothrombosis for Continued Health (REACH) Registry was an international, prospective cohort study of 68,236 patients with either established atherosclerotic arterial disease or at least three risk factors for atherothrombosis, who were enrolled from 5587 physician practices in 44 countries [10]. Among those with CeVD alone, at 1 year, the risk of cardiovascular death, myocardial infarction, stroke, or hospitalisation for atherothrombotic events was 9.87% (95% CI 9.24–10.50), but it more than doubled to 21.95% (95% CI 19.43–24.40) among those with both CeVD and PAD. A meta-analysis of 11 studies (n = 5374) showed that a low ankle–brachial index (ABI), a measurement of PAD, was associated with both an increased hazard of recurrent stroke (hazard ratio (HR) 1.70, 95% CI 1.10–2.64) and an increased risk of vascular events or vascular death (HR 2.22, 95% CI 1.67–2.97) [11]. It is thus imperative that PAD is suspected, diagnosed and treated among those with IS to reduce the risk of subsequent adverse events.

### 1.4. PAD Among Patients with Cerebrovascular Disease, Knowledge Gaps

Studies involving patients with acute IS (AIS), some of which also included patients with transient ischaemic attacks (TIAs), reported a prevalence of PAD based on ABIs ≤ 0.9 that ranged widely, from 6 to 51%; an earlier meta-analysis reported a range of 7.4–40.5% [12]. Most of these studies were in Western populations. These include, in ascending order, the United Kingdom (6.0%) [13], Greece (14.8%, 19.8%, 24.6%) [14,15,16], Spain (24.1%, 40.5%) [17,18], the United States (26%) [19], Germany (31%, 32%, 51%) [20,21,22], Italy (33.5%) [23], Austria (44.9%) [24], and Poland (46%) [25]. There was a multi-centre study that included 17 low-income and middle-income countries in Latin America, the Middle East, North Africa, and South Africa that reported a prevalence of 22.3% [26]. In the international REACH registry, approximately 11% of patients with IS had PAD [27]. However, there is a knowledge gap, as there are few Asian studies. More data is needed from Asia to provide a more balanced picture of PAD among IS patients globally. Also, Asia is home to two-thirds of the world’s population, and with a disproportionate burden of stroke [1]. If there is an added burden of PAD in Asia, additional resources will be needed to diagnose and treat both conditions, in addition to the vascular risk factors and other co-morbidities. This can be a challenge in resource-limited settings. It is also unclear which patients with IS are more likely to have PAD. Having the relevant clinical information would be helpful to the managing team to focus their PAD-screening investigations on appropriate patients and, after detection, to subsequently provide individualised optimal therapy.

### 1.5. Study Aim

This cross-sectional study was thus performed with the aim of determining the prevalence, risk factors and clinical markers for PAD among acute IS patients in Singapore, a country in Asia.

## 2. Materials and Methods

### 2.1. Study Site

The study was performed in Tan Tock Seng Hospital, a publicly funded restructured hospital located in central Singapore that caters to the health needs of the surrounding population of 1.4 million people.

### 2.2. Inclusion and Exclusion Criteria

This report is a further analysis of a study of PAD among high-risk patients, focusing on those admitted for AIS [28]. Consecutive patients admitted for AIS to the Department of Neurology were approached to participate. Inclusion criteria for the study were: 1. Clinical diagnosis of stroke of rapidly developing clinical signs of focal (at times global) disturbance of cerebral function lasting more than 24 h or leading to death with no apparent cause other than that of vascular origin [29] 2. Brain imaging was performed by either Computed Tomography (CT) or Magnetic Resonance Imaging (MRI). 3. Brain imaging revealed an ischaemic lesion consistent with the clinical syndrome. Stroke was also diagnosed if there were global or focal neurological symptoms lasting <24 h, but imaging evidence of ischaemic brain tissue injury attributable to a vascular cause was found [30] 4. admitted within 1 week of onset of stroke symptoms. Exclusion criteria were: 1. Aphasic or drowsy throughout the stay in hospital. 2. Amputations of both upper limbs or both lower limbs. 3. Unable to obtain informed consent.

### 2.3. Data Collection

**Figure 1:**
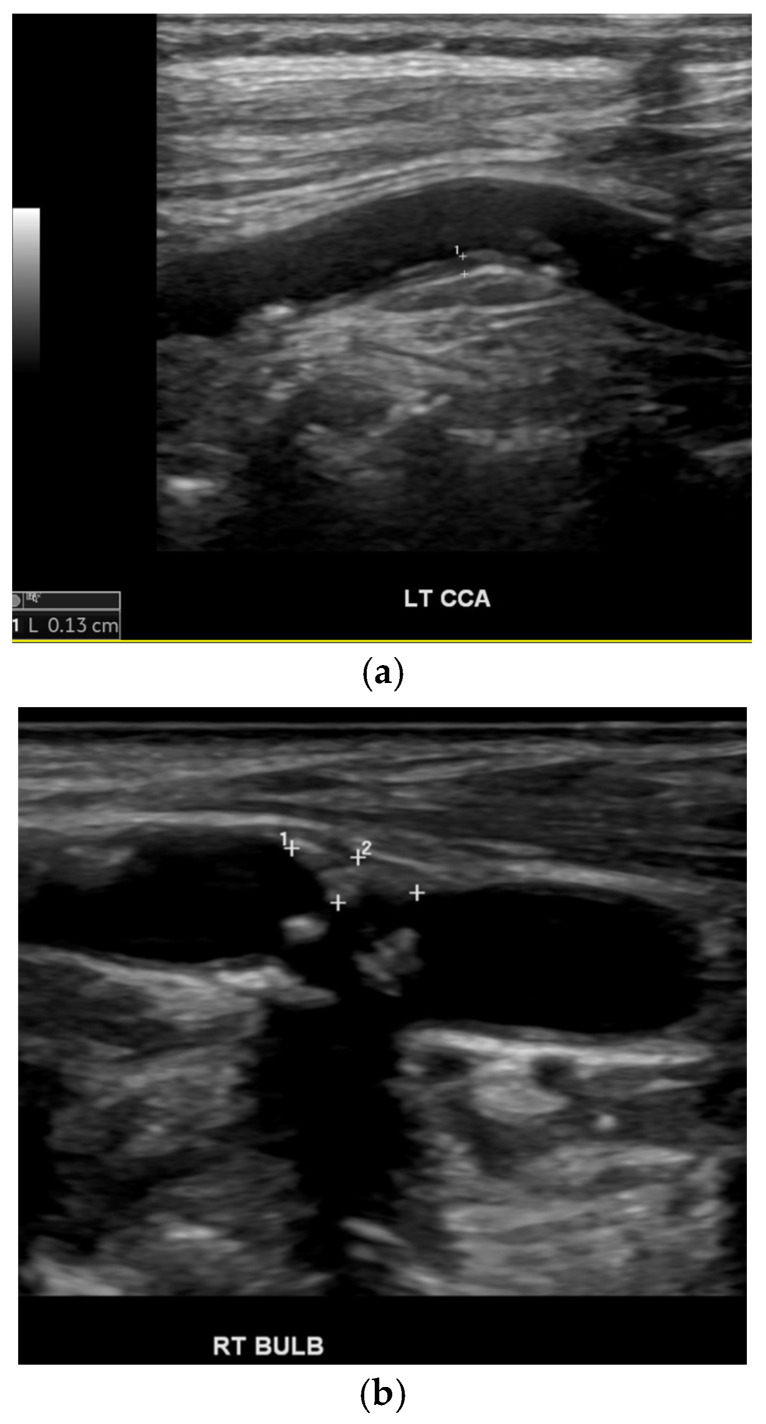
(**a**) Intima–medial thickening of the left common carotid artery. (**b**) Plaques in the right carotid bulb.

**Figure 2:**
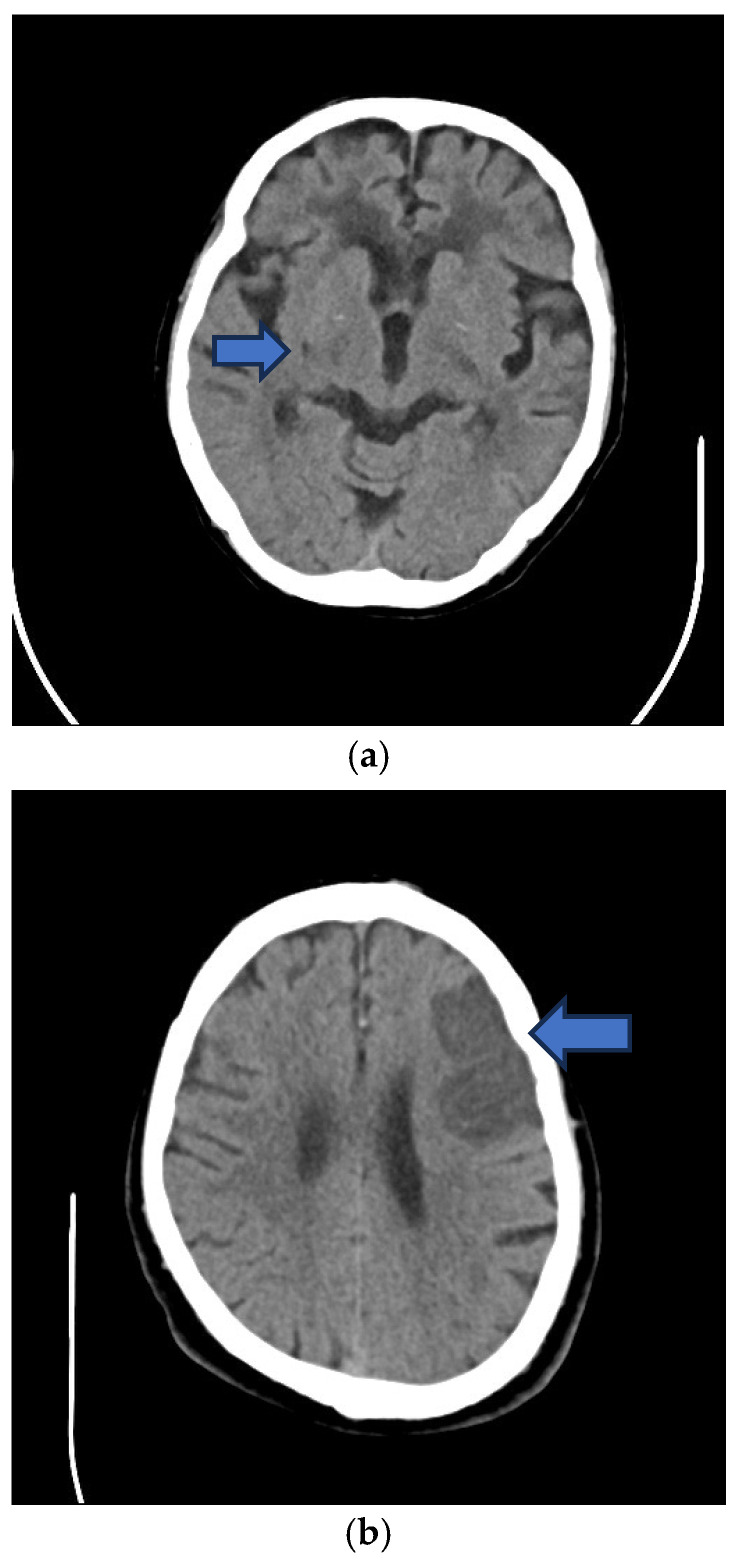
(**a**) Lacunar infarct in right internal capsule. (**b**) Non-lacunar infarct in left frontal lobe.

After informed consent was obtained from eligible subjects or from their legally acceptable representatives, data was collected by the trained study nurse using a standardised questionnaire. Data included subject demographics (sex, age). Hypertension was diagnosed if there was a history of hypertension or if the patient had been prescribed medications to lower blood pressure. DM was diagnosed if there was a history of DM or if the patient had been prescribed medications to lower glucose. Hypercholesterolaemia was diagnosed if there was a history of hypercholesterolaemia or if the patient had been prescribed medications to lower lipids. Cigarette smoking was diagnosed if the patient had ever smoked a cigarette. Ischaemic heart disease (IHD) was diagnosed if there was a history of angina pectoris, myocardial infarction, or coronary artery interventions. Body mass index (BMI = weight/height^2^) was calculated. The presence of carotid intima–medial thickening (IMT ≥ 1.0 mm) (Figure 1a) and carotid plaques (CP) (Figure 1b) and the degree of stenosis were determined by Duplex carotid ultrasonography. IS was classified as lacunar (LI) (Figure 2a) or non-lacunar (NLI) (Figure 2b) based on brain imaging; LI was defined as small, deep cerebral infarct measuring 2 to 20 mm in diameter due to occlusion of a small artery/penetrator attributed to ageing, hypertension and arterial lipohyalinoiss or microthrombosis and associated with the clinical lacunar syndrome of pure motor stroke, pure sensory stroke, sensory motor stroke or ataxic hemiparesis–dysarthria clumsy hand syndrome [31]; all other infarcts were categorised as NLI, which may be due to atherothrombosis, cardioembolism, uncommon arterial wall pathologies, and rarely hypercoagulability.

### 2.4. Diagnosis of PAD

The ABI was determined in the left and right leg with the subject lying in the supine position; the highest systolic blood pressure (SBP) in the posterior tibial or dorsalis pedis artery of that lower limb, determined using the Doppler probe, was divided by the highest SBP in both brachial arteries using the standard auscultatory technique. The research staff performing ABI measurements were kept blinded to other study information. PAD was diagnosed if ABI was ≤0.9 in either limb; this cut-off has a sensitivity of 61% (95% CI 55–69%) and specificity of 92% (95% CI 89–95%) [32,33].

### 2.5. Data Analysis

Data were analysed using the Statistical Package for Social Sciences (SPSS) v25 (New York, NY, USA). Means and standard deviations were calculated for normally distributed continuous variables, median and interquartile ranges for non-normally distributed continuous variables, and proportions for categorical variables. Significant differences in the baseline characteristics were evaluated among those with and without PAD via the univariable analysis, using the unpaired *t*-test for continuous variables and a chi-square test for categorical variables. Subsequently, a multivariable analysis using logistic regression was first performed using demographic and risk factor variables (Model 1), then after adding carotid Duplex findings (Model 2), and finally after also adding the brain imaging findings (Model 3). Statistical significance was taken at the *p* ≤ 0.05 level.

### 2.6. Sample Size Estimation

Using the estimate of a prevalence of PAD among IS patients as 11% based on the international REACH registry [10], and a 95% confidence interval with a margin of error of 5%, the sample size would be 151 subjects.

### 2.7. Ethics

This study was approved by the institutional Ethics Committee of Tan Tock Seng Hospital, Singapore, protocol number CR/ETHICS/no funding/353 on 17 January 2000, before the study was started. The study was conducted in accordance with the principles set forth in the Helsinki Declaration. Patients or their legally acceptable representatives gave their signed informed consent for participation in the research study after reading the patient information sheet and having their questions answered to their satisfaction.

## 3. Results

### 3.1. Subject Demographics

A total of 150 subjects were recruited out of 153 screened subjects (3 excluded as they remained drowsy and aphasic throughout their hospital stay, and consent was not obtainable); the mean age was 62.7 ± 10.2 years, 44.7% were female, and the mean BMI was 24.1 ± 4.1 (Table 1). Hypertension was reported in 63.3%, DM in 42.7%, hypercholesterolaemia in 30.0%, smoking in 38.0%, previous stroke in 18.7%, and IHD in 6.0% of patients. IMT was detected in 30.7%, CP in 77.3%, and carotid stenosis ≥50% in 8.0%. LI occurred in 64.7%, NLI in 35.3%.

### 3.2. Frequency of PAD

PAD was diagnosed in 22.0% (95% CI 16.1–29.3) based on ABI.

### 3.3. Analysis of Factors Associated with PAD

On univariate analysis, based on demographics and vascular risk factors, PAD was significantly associated with age (*p* = 0.03), hyperlipidaemia (*p* = 0.03), and IHD (*p* = 0.004). On logistic regression (model 1), PAD was significantly associated only with IHD (aOR = 6.42, 95% CI 1.25–32.84; *p* = 0.03). When carotid IMT and carotid plaque were added to the model (model 2), only the association with IHD remained (aOR = 5.45, 95% CI 1.01–28.71; *p* = 0.045). When the result of brain imaging was added (model 3), the significant association was only with NLI (aOR = 2.78, 95% CI 1.09–7.14; *p* = 0.03) (Table 1).

## 4. Discussion

### 4.1. Summary of Findings

In this study of patients hospitalised with acute IS in Singapore, PAD based on ABI was detected in 22.0%. Based on vascular risk factors alone, PAD was associated with increasing age, hypercholesterolaemia and IHD. On logistic regression, it was only associated with IHD, an association that remained after carotid IMT and plaque were added to the model. However, when the results of brain imaging were added, the association was only with non-lacunar infarction.

### 4.2. Comparison with Other Studies—Frequency of PAD

The prevalence of 22% in this study can be compared with other Asian studies of PAD in IS. These showed a range of 7.4% to 58%. These included, generally in ascending order, Korea (7.4%, 9.9%, 10.1%, 13%) [34,35,36,37], Japan (11.5%, 18.8%) [38,39], China (17.7%) [40], Thailand (18.1%) [41], Pakistan (18.3%) [42], India (24.3%, 29.2% IS and haemorrhagic stroke HS combined, 58%) [43,44,45], Bangladesh (26%) [46], Singapore (26.2%) [47], and Malaysia (28.3%) [48]. The findings in this study are thus within the ranges reported elsewhere in Asia, and the 6–51% reported from studies performed in predominantly Western populations [12].

### 4.3. Comparison with Other Studies—Demographics

The patient demographics in this study, with respect to age being in the 60s and fewer females than males, are also consistent with other Asian PAD–stroke studies (Table 2). This finding has also been reported in other stroke studies in Asia [49]. The lower incidence of stroke in females compared to males [1] may be due to a lower frequency of vascular risk factors and the vascular protective effect in females of endogenous oestrogens [50].

### 4.4. Comparison with Other Studies—Vascular Risk Factors

Vascular risk factor profiles of the study patients also resemble other Asian studies in general [50], as well as PAD–stroke studies, with hypertension being the most common risk factor, but with differing frequencies of DM, hyperlipidaemia, smoking, previous stroke, and IHD (Table 2). This variability in risk factor profiles in differing populations is well-described [1], with some populations having more of some risk factors compared to others, e.g., the known particularly high prevalence of diabetes mellitus in South Asian countries [51], which was also seen in this study.

### 4.5. Comparison with Other Studies—Factors Associated with PAD

This study showed an association of PAD with older age, hyperlipidaemia and IHD in the univariable analysis; however, on logistic regression, the association only remained with IHD. Other Asian studies also found various associations in the univariable analysis (UA) (Table 2), but among those that performed logistic regression, an association with IHD was also reported in Bangladesh (compared to stroke-free controls) [46] and Thailand [41]. The only other study that performed a logistic regression test was 1 of the 3 studies from Korea [37]; the others only reported univariable analyses. Still, there was no consistent pattern of associations seen across all studies. This variability, if true, may reflect the unique situation of each country.

A possible surrogate for PAD is carotid arterial disease. This study did find an association in the univariable analysis between PAD and carotid IMT, but not with the presence of carotid plaques. An association with carotid disease was also reported in Bangladesh [46], Japan [38] and Singapore [47]. However, a multivariable analysis was only performed in this study and the Bangladesh study; the former involved comparison with stroke-free controls, while the latter only involved stroke patients and did not find an association. Thus, the value of using the presence of carotid disease as a surrogate for PAD in Asian populations remains uncertain. However, in other studies’ univariable analyses, associations were found with cerebral atherosclerosis (Korea) [36], intracranial stenosis (Japan) [38], intracranial large-artery disease (Singapore) [47] and large-artery atherosclerosis (Korea) [37]; only the latter involved a multivariable analysis.

The above findings are possibly indicative that PAD is associated with atherosclerosis involving the large cranio-cerebral arteries (large-artery atherosclerosis (LAA)). A disease of the arteries would cause non-lacunar infarcts. Lacunar infarcts are small, deep cerebral infarcts, from 2 to 20 mm in diameter [31], due to intracranial small vessel occlusions (SVOs) from lipohyalinosis, which are attributable to ageing and hypertension [52], though microatherosclerosis–atherothrombosis may also play a part. All other (and thus larger) infarcts may be categorised as non-lacunar, with varying mechanisms, especially atherosclerosis, with cardioembolism being much less frequent. The study from Korea did show a greater association between PAD and LAA compared to SVO [37], while the study from Japan also showed an association with non-SVO stroke [38]. A study from China had also detected PAD in 31.51% of patients with LAA and 19.75% in small artery disease [53]. These are consistent with the findings of this study of the association between PAD and non-lacunar stroke. This is of clinical value; clinicians managing IS patients with non-lacunar infarcts may thus wish to especially investigate for PAD in these patients by a simple bedside test—the ABI.

### 4.6. Clinical Relevance of PAD in IS Patients

It is medically important to detect PAD in patients with IS. As mentioned earlier, patients with IS and PAD have a worse prognosis compared to those with IS, but no PAD; they have an increased risk of subsequent major atherothrombotic events, including recurrent stroke, myocardial infarction, cardiovascular death or hospitalisation for atherothrombotic events [10,11]. Thus, the patient and family need to be appropriately counselled. Secondly, those with IS and PAD have a doubling of the 1-year cost of care for hospitalisation [54]. This makes it imperative that PAD be actively looked for in patients with IS and be treated appropriately. Thirdly, while aspirin and clopidogrel may be considered equivalent in efficacy in secondary prevention after IS, the superiority of clopidogrel over aspirin among patients with PAD [55] suggests that clopidogrel may be the preferred antithrombotic over aspirin among those with both IS and PAD, to reduce the risk for subsequent vascular events. Fourthly, while PAD needs to be managed in its own right [56,57,58], PAD may worsen limb function already adversely affected by the stroke, hampering rehabilitation and recovery after stroke—a low ABI reduces mobility [59]. All these suggest that PAD should be looked for in patients with IS so as to enable holistic care to be provided.

### 4.7. Study Limitations

There are a few study limitations. This is a single-centre study, which may affect generalisability. The relatively small patient numbers may lead to reduced power to detect clinically important differences. Duration of the vascular risk factors, severity, adequacy of control, treatment intensity and the medications used were not evaluated; smoking was categorised as never or ever smoked; these may have an impact on the development of PAD. Patients who were aphasic or drowsy, which may be due to strokes from large artery occlusion were excluded; in view of the findings of this and other studies—that it is these patients who may be more likely to have PAD—the prevalence of PAD in this study may have been under-estimated, but as only 3 patients were excluded for this reason, the overall results are unlikely to have been affected. The logistic models included multiple covariates despite there being only 33 PAD events. That is a difficult ratio for stable multivariable analysis. The shift from IHD in one model to NLI when added to the final model suggests model sensitivity. Stroke was divided into lacunar and non-lacunar infarction on imaging, rather than using a more detailed etiologic classification throughout the study population. Non-lacunar infarction is a broad category and does not map cleanly to large-artery atherosclerosis. With regard to data on the infarction size or by the clinical grading system, volumetric determination of the infarct size was not performed, and the stroke severity by the National Institute of Health Stroke Scale (NIHSS) was not consistently recorded and thus unavailable for analysis. Finally, as this is a cross-sectional study, it cannot determine whether PAD predicts stroke subtype, future vascular events, or treatment response.

Still, this study has some strengths. The patients were well-characterised with all clinically important data considered. The findings are consistent with other studies, which makes the findings believable. Detailed statistical analysis was performed with logistic regression, which allows adjustment for the effects of confounders, which was not performed by most other PAD-IS studies.

## 5. Conclusions

This study shows a high prevalence of PAD among Asian patients with acute IS. It is associated with non-lacunar infarctions. Further studies with larger patient numbers involving multiple centres with detailed statistical analysis are needed to corroborate these findings.

## Figures and Tables

**Table 1:** Characteristics of study patients.

Characteristic	Overall	Peripheral Artery Disease			Model 1			Model 2			Model 3		
	n = 150	Y (n = 33)	N (n = 117)	*p*-Value	aOR	95% CI	*p*-Value	aOR	95% CI	*p*-Value	aOR	95% CI	*p*-Value
Age (years, mean ± SD)	62.7 ± 10.2	66.1 ± 11.1	61.7 ± 9.7	0.03	1.04	0.99–1.08	0.13	1.03	0.98–1.08	0.26	1.04	0.99–1.09	0.13
Female (%)	44.7	48.5	43.6	0.38	1.01	0.36–2.86	0.99	1.32	0.43–4.04	0.63	1.22	0.39–3.84	0.73
Body Mass index (BMI) (kg/m^2^)	24.1 ± 4.1	23.3 ± 3.8	24.4 ± 4.1	0.20	0.96	0.86–1.08	0.49	0.98	0.87–1.11	0.78	0.97	0.85–1.10	0.62
Hypertension (%)	63.3	66.7	62.4	0.41	0.77	0.30–1.97	0.59	0.61	0.22–1.65	0.33	0.54	0.19–1.50	0.24
Diabetes mellitus (%)	42.7	54.5	39.3	0.09	2.34	0.95–5.78	0.07	1.58	0.62–4.08	0.34	1.43	0.54–3.78	0.47
Hypercholesterolaemia (%)	30.0	45.5	25.6	0.03	1.77	0.72–4.38	0.21	1.87	0.73–4.81	0.20	2.08	0.78–5.53	0.14
Ever smoked (%)	38.0	39.4	37.6	0.50	1.26	0.44–3.59	0.66	1.25	0.43–3.65	0.69	1.22	0.41–3.63	0.73
Previous stroke (%)	18.7	24.2	17.1	0.24	1.11	0.39–3.14	0.85	1.01	0.35–2.95	0.99	1.01	0.34–3.00	0.99
Ischaemic heart disease (%)	6.0	18.2	2.6	0.004	6.42	1.25–32.84	0.03	5.45	1.03–28.71	0.045	4.53	0.86–23.77	0.07
Intima-medial thickening (%)	30.7	39.4	28.2	0.16				1.64	0.64–4.18	0.30	1.94	0.73–5.15	0.18
Carotid plaque (%)	77.3	97.0	71.8	0.001				7.85	0.94–65.37	0.06	8.28	0.96–71.20	0.054
Non-lacunar infarct (%)	35.3	48.5	31.6	0.06							2.78	1.09–7.14	0.03

Legend: Y = yes; N = no; aOR = adjusted odds ratio; CI = confidence interval; SD = standard deviation.

**Table 2:** Comparative studies of peripheral arterial disease in stroke patients in Asia.

Country	Author, Year	N (IS/TIA/HS)	PAD (%)	Age (yr)	F (%)	BMI (kg/m^2^)	HT (%)	DM (%)	HL (%)	SM (%)	Stroke (%)	IHD (%)	CD (%)	SVO, LI (%)	Significant Factors for PAD
Bangladesh	Shahi, 2013 [46]	50/ 0/0	26.0	62.3 + 7.5	34.0	(>23) 42	76.0	52.0	42.0	74.0	8.0	20.0	32	NR	(cf SFC, LR) Stroke aOR 3.91 (95% CI 1.87–33.18, *p* = 0.002) IHD aOR 3.00 (95% CI 3.05–44.32, *p* = 0.005) CD aOR 2.46 (95% CI 1.68–15.31, *p* = 0.026)
India	Thakare, 2015 [44]	88/0/32	29.2	57.5	26.7	NR	54.2	45.0	NR	26.7	29.2	22.5	NR	NR	UA Age 70–79 yr (*p* < 0.0001) Stroke OR 2.43 (95% CI 1.06–5.62, *p* = 0.04)
Japan	Hoshino, 2013 [39]	68/25/8	18.8	NA	19.9	NA	79.2	32.7	68.3	8.9	6.9	9.9	NR	14.3	UA Age 75.8 + 7.8, 76.8 + 8.8 vs. 70.6 + 9.8 (*p* = 0.02) DM 4.98 (95% CI 1.73–14.31, *p* < 0.01)
Japan	Ishizuka, 2014 [38]	209/ 0/0	11.5	67.7 + 12.6	31.6	NR	73.2	40.2	50.7	16.7	NA	16.7	Stenosis 21.1	29.2	UA CD OR 2.57 (95% CI 1.04–6.35, *p* = 0.04) Intracranial stenosis OR 5.06 (95% CI 2.09–12.25, *p* = 0.0003) TOAST LAA OR 3.31 (95% CI 1.39–7.90, *p* = 0.007) TOAST non-SVO OR 3.49 (95% CI 1.33–9.16, *p* = 0.01)
Korea	Lee, 2012 [34]	1147/ 0/0	7.4	64.5 + 12.3	39.8	23.8	74.7	31.2	13.7	40.9	NR	19.3	NR	10.5	UA Age 70.5 + 11.8 vs. 64.0 + 12.2 (*p* < 0.001) BMI 22.8 + 2.8 vs. 23.9 + 3.2, (*p* = 0.001) DM OR 1.60 (95% CI 1.02–2.52, *p* = 0.04) IHD OR 2.35 (95% CI 1.46–3.77, *p* < 0.001)
Korea	Kim, 2012 [36]	775/ 0/0	10.1	65.5 + 12.5	37.5	23.8 + 3.2	72.9	31.1	23.7	26.3	NR	20.1	NR	NR	UA Age 71.7 + 11.7 vs. 64.8 + 12.4, (*p* < 0.001) BMI 22.48 + 2.6 vs. 23.9 + 3.2, (*p* < 0.001) HT OR 2.74 (95% CI 1.38–5.42, *p* = 0.002) DM OR 1.94 (95% CI 1.21–3.12, *p* = 0.007) IHD OR 2.18 (95% CI 1.31–3.62, *p* = 0.004) Cerebral atherosclerosis OR 2.34 (95% CI 1.41–3.90, *p* = 0.001)
Korea	Chung, 2013 [37]	1182/ 111/0	13.0	65.5 + 12.4	41.4	NR	63.8	31.4	42.6	28.2	18.6	6.5	NR	27.5	LR Age aOR 1.05/yr (95% CI 1.03–1.05, *p* < 0.001) DM aOR 1.53 (95% CI 1.04–2.27, *p* = 0.03) HL aOR 2.87 (95% CI 1.97–4.17, *p* < 0.001) TOAST LAA aOR 2.32 vs. SVO (95% CI 1.37–3.93, *p* < 0.01)
Pakistan	Rahman, 2017 [42]	327/ 0/0	18.3	57.6 + 12.8	48.6	(>23) 42	91.7	57.8	18.7	17.1	15.3	24.5	100	8	UA HT OR 0.28 (95% CI 0.12–0.65, *p* = 0.003)
Singapore	Manzano, 2012 [47]	1311/ 0/0	26.2	NA	43.1	NR	78.3	40.8	56.6	25.6	NR	23.8	>70% stenosis 5.7	NR	UA Female sex OR 2.08 (95% CI 1.61–2.67, *p* < 0.0001) HT OR 1.58 (95% CI 1.14–2.19, *p* = 0.006) DM OR 1.72 (95% CI 1.35–2.19, *p* < 0.0001) Severe CD OR 2.10 (95% CI 1.35–3.55, *p* = 0.001) ICLAD OR 1.77 (95% CI 1.38–2.28, *p* < 0.0001)
Thailand	Ratanakorn, 2012 [41]	694/ 53/0	18.1	63.5 + 14	44.2	NR	59.4	30.9	81.8	17.3	14.7	9.6	>50% 3.2	39.0	LR Age ≥ 60 yr aOR 3.54 (95% CI 2.14–5.85, *p* < 0.001) Female sex aOR 1.61 (95% CI 1.09–2.40, *p* = 0.02) Stroke aOR 2.15 (95% CI 1.32–3.49, *p* = 0.002) IHD aOR 2.55 (95% CI 1.4 7–4.43, *p* = 0.01) Atrial fibrillation aOR 1.71 (95% CI 1.03–2.82, *p* = 0.04)
Singapore	This study	150/ 0/0	22.0	62.7 + 10.2	44.7	24.1 (4.1)	63.3	42.7	30.0	38.0	18.7	6.0	IMT 20.7, CP 77.3	64.7	LR Non-lacunar aOR 2.78 (95% CI 1.09–7.14, *p* = 0.03)

(Legend: N = number; IS = ischaemic stroke; TIA = transient ischaemic attack; HS = haemorrhagic stroke; PAD = peripheral arterial disease; F = female; BMI = body mass index; HT = hypertension; DM = diabetes mellitus; HL = hyperlipidaemia; SM = smoking; IHD = ischaemic heart disease; CD = carotid disease; SVO = small vessel occlusion; LI = lacunar infarction; SFC = stroke-free controls; LR = logistic regression; UA = univariable analysis; TOAST = trial of Organon in acute stroke treatment classification; LAA = large-artery atherosclerosis; ICLAD = intracranial large-artery atherosclerosis).

## Data Availability

Data are available from Tan Tock Seng Hospital.
